# BFD2 mediates inflammation, apoptosis, and pre-anxiety-like behaviors induced by acute *Toxoplasma gondii* infection

**DOI:** 10.1371/journal.pntd.0013428

**Published:** 2025-09-04

**Authors:** Xiaocheng Zhang, Tanzhen Xu, Jinjin Zhu, Hui Peng, Zixin Wei, Lijun Cui, Qingqiu Zuo, Hua Liu, Yuan Hu, Jianping Cao

**Affiliations:** 1 National Key Laboratory of Intelligent Tracking and Forecasting for Infectious Diseases, Beijing, China; 2 Key Laboratory of Parasite and Vector Biology, National Health Commission of the People’s Republic of China, Shanghai, China; 3 World Health Organization Collaborating Centre for Tropical Diseases, Shanghai, China; 4 National Institute of Parasitic Diseases at Chinese Center for Disease Control and Prevention (Chinese Center for Tropical Diseases Research), Shanghai, China; 5 The School of Global Health, Chinese Center for Tropical Diseases Research, Shanghai Jiao Tong University School of Medicine, Shanghai, China; 6 Department of Clinical Pharmacy, Fuyang Cancer Hospital, Fuyang, China; 7 Department of Biochemistry and Molecular Biology, School of Basic Medical Sciences, Anhui Medical University, Hefei, China; 8 Shanghai Municipal Center for Disease Control and Prevention, Shanghai, China; Ege Universitesi Tip Fakultesi, TÜRKIYE

## Abstract

*Toxoplasma gondii* infection induces anxiety in hosts during the chronic stage; however, its role in pre-anxiety-like behaviors during the acute stage remains poorly understood. This study investigates the role of Bradyzoite Formation Deficient 2 (BFD2), a transcription factor essential for tachyzoite-to-bradyzoite differentiation, in inflammation, apoptosis, and behavioral changes during acute *T. gondii* infection. Using CRISPR/Cas9-mediated gene editing, we generated a *Bfd2* knockout strain (ME49∆*bfd2*) and observed reduced parasite proliferation and plaque formation, indicating BFD2’s role in promoting *T. gondii* survival. RNA sequencing analysis of infected BV2 cells revealed that *Bfd2* deletion significantly downregulated inflammatory responses, with reduced expression of key inflammatory markers (interleukin 1 beta ((IL-1β), interferon gamma (IFN-γ), and tumor necrosis factor alpha (TNF-α)) during acute infection. Next, we used western blotting, real-time quantitative PCR (qPCR) and enzyme-linked immunosorbent assays (ELISAs) to verify that BFD2 improves the inflammation induced by acute stage *T. gondii* infection. *In vivo* studies confirmed that BFD2 exacerbates brain inflammation and neuronal apoptosis specifically during the acute stage, with no significant effects during the chronic stage. Behavior was assessed using the elevated plus maze test and open field test. Compared with the uninfected group and ME49∆*bfd2* group, the ME49 group mice showed an increased percentage of distance in the open arms and time in the open arm. The results showed that the total distance traveled, distance in the center, and time in the center were significantly decreased in the ME49 group, and the total distance traveled (mm) had no significant changes in the ME49∆*bfd2*. These demonstrated that BFD2 contributes to pre-anxiety-like behaviors in mice during acute stage *T. gondii* infection. These findings highlight BFD2 as a critical regulator of acute-stage inflammation, neuronal damage, and behavioral alterations, providing insights to develop targeted interventions against *T. gondii* infection.

## Author summary

Acute-to-chronic stage conversion in *Toxoplasma gondii* differentiation depends on the transcriptional changes induced by bradyzoite formation deficient 2 (BFD2). However, the potential roles of inflammation, apoptosis, and pre-anxiety-like behaviors in the acute stages *T. gondii* infection remain unclear. Here, we found that *T. gondii* acute infection induced pre-anxiety-like behaviors, characterized by BFD2-mediated brain inflammation and apoptosis in mice. Thus, we revealed the effect of BFD2 in mediating the anxiety-like behaviors induced during acute *T. gondii* infection. Overall, these findings provide new insights for the development of vaccines against *T. gondii*-related infections.

## Introduction

*Toxoplasma gondii*, a cosmopolitan protozoan parasite, infects nucleated cells across all warm-blooded species, including humans [1]. During pregnancy, infection with *Toxoplasma gondii* might cause fetal malformations, with severe cases leading to spontaneous abortion or even stillbirth [2]. It is widely accepted that pathological manifestations primarily occur in fetuses or immunocompromised individuals, whereas immunocompetent hosts typically maintain the parasite as a chronic, subclinical, latent cerebral infection without overt symptoms [3]. However, recent research has confirmed that *T. gondii* infection induces sustained inflammatory responses, which might lead to increased anxiety-like behaviors in hosts and is considered a potential risk factor for anxiety and related neuropsychiatric disorders [4,5]. Acute infection with the parasite is mainly characterized by an inflammatory response, in which interferon-dependent immune responses control rapid parasite expansion and mitigate acute disease symptoms, with chronic infection in the central nervous system being caused by the existence of cysts in the brain or other organs of the host [6–8]. While *T. gondii* replication increases parasite transmission, the resultant inflammatory reaction drives the host immune response to promote pathogen elimination, which leads to immune suppression [9]. Many protozoan parasites have evolved to include slow-growing chronic stages that can reduce immunogenicity [10].

For apicomplexans like *T. gondii*, the chronic stage of infection is the result of intracellular parasitism of bradyzoites. While infected hosts can harbor these cysts and survive in the long-term, the proliferation of tachyzoites can lead to serious and potentially fatal diseases. The differentiation of tachyzoites (acute stage) into bradyzoites (chronic stage) can thereby promote the pathological effect on the host and ensure parasite transmission. Bradyzoite Formation Deficiency 1 (BFD1) is a Myb like transcription factor that plays a critical role in the chronic differentiation of *T. gondii* [11]. Bradyzoite Formation Deficient 2 (BFD2), CCCH-type zinc finger family protein, is one of five factors that are transcriptionally activated by BFD1 [12]. In recent years, the effects of chronic *T. gondii* infection on mental disorders (anxiety-like behaviors) have been reported [13–16]. However, it is unclear whether anxiety-like behaviors are induced by acute-stage *T. gondii* infection.

The present study aimed to determine whether acute-stage *T. gondii* infection induces anxiety-like behaviors and whether this involves BFD2. The results showed that BFD2, besides regulating tachyzoite differentiation, can contribute to anxiety-like behaviors induced by acute *T. gondii* infection. Overall, these findings revealed a crucial role of BFD2 in mediating *T. gondii*-induced anxiety, and will provide the basis for developing BFD2-based vaccines against *T. gondii*.

## Methods

### Ethics Statement

All animal experiments were designed to minimize pain and harm to the greatest extent possible, and were performed in strict accordance with the Regulations for the Administration of Affairs Concerning Experimental Animals (approved by the State Council of People’s Republic of China). All procedures performed on animals in this study were approved by the Laboratory Animal Welfare & Ethics Committee (LAWEC) of the National Institute of Parasitic Diseases, Chinese Centre for Disease Control and Prevention (Chinese Center for Tropical Diseases Research; approval ID: IPD-2021–18).

### Reagents

Human foreskin fibroblasts (HFFs) and mouse microglia (BV2) cells were purchased from American Type Culture Collection (ATCC, Manassas, VA, USA). The pSAG1::CAS9-U6::sgUPRT and pUPRT::DHFR-D plasmids were purchased from Addgene (Cambridge, MA, USA). Top 10 competent *Escherichia coli* cells (CB104), the EndoFree Maxi plasmid Kit (DP117), and the TIANcombi DNA Lyse&Det PCR Kit (KG203) were purchased from Tiangen Biotech Co., Ltd. (Beijing, China). The Easy Pure Genomic DNA Kit was purchased from TransGen Biotech Co., Ltd (Beijing, China). The Q5 Site-Directed Mutagenesis Kit (E0552S) was purchased from NEB (Ipswich, MA, USA). The ReverTra Ace qPCR RT Kit (FSQ-101) and the SYBR RT-PCR kit (QPK-212) were purchased from Toyobo (Osaka, Japan). Ampicillin (100 mg/mL solution) was purchased from Solarbio (Shanghai, China). T4 DNA Ligase (15224017) and TRNzol (15596018CN) were purchased from Invitrogen (Carlsbad, CA, USA). Dulbecco’s Modified Eagle Medium (DMEM), Dulbecco’s Phosphate-Buffered Saline (DPBS), and fetal bovine serum were purchased from Gibco (Carlsbad, CA, USA). We acquired 4 mm cuvettes (1652081) from Bio-Rad Laboratories, Inc. (Hercules, CA, USA). Anti-nuclear factor kappa B (NF-κB) p65 (8242), Anti-Phospho-NF-κB p65 (3033), Anti-signal transducer and activator of transcription 3 (Stat3) (9139) and Anti-Phospho-Stat3 (9145) antibodies were purchased from Cell Signaling Technology (Danvers, MA, USA).

### Parasites and animals

*T. gondii* ME49 was preserved by the Institute of Parasitic Disease Prevention and Control, Chinese Center for Disease Control and Prevention. Specific-pathogen-free female wild-type C57BL/6 mice (6–8 weeks old), were purchased from the Shanghai Linchang Laboratory Animal Co., Ltd. (Shanghai, China). All mice were housed in the specific-pathogen-free animal facilities of the Institute of Parasitic Disease Prevention and Control, Chinese Center for Disease Control and Prevention.

### *T. gondii* culture

*T. gondii* were cultured and maintained in HFFs at 37°C under 5% CO_2_ in standard medium, consisting of DMEM supplemented with 5% fetal bovine serum, 2 mM L-glutamine (Beyotime, Shanghai, China), and 10 μg/mL ampicillin. *T. gondii* electroporation was performed in 4 mm cuvettes using a wave electroporator and subcloning was performed in 96-well plates by sorting or plating using limiting dilution, with strains verified by at least two rounds of genotyping-PCR and sequencing [12].

### The construction of BFD2-knockout plasmids and ∆*bfd2* knockout strains

Briefly, a single guide RNA (sgRNA) for *T. gondii BFD2* was designed using the E-CRISPR website http://www.e-crisp.org/E-CRISP/. Using pSAG1::CAS9-U6::sgUPRT as the template and the designed SgRNA-F and SgRNA-R as primers, the Q5 Site-Directed Mutagenesis Kit was used to mutate sgUPRT in the template plasmid into the sgRNA of *T. gondii BFD2*. The mutated plasmid was transformed into Top 10 competent *E. coli*. Sequencing further verified the correct positive plasmid, which was named it pSAG1::CAS9-U6::sgBFD2. Using pUPRT::DHFR-D as a template and *bfd2*-F and *bfd2*-R as primers, we amplified the pyrimethamine resistance gene with 40 bp homology to regions immediately up- and downstream of *BFD2*. We used PCR to verify both the integration of *BFD2* and reciprocal loss of the wild-type untagged allele.

Then, 25 μg each of the pSAG1::CAS9-U6::sgBFD2 and donor DNA in a 2:1 ratio were transfected into ME49 parasites to generate the Δ*bfd2* strain by electroporation under conditions of 1500 V, 50 μF, 50 Ω,176 μs, 100 ms interval (carried out twice). After electroporation, the tachyzoites were inoculated into HFF cells and cultured for 24 hours. During the cultivation process, the DMEM medium containing 3 μM ethylamine pyrimidine and 2% fetal bovine serum was replaced every 1–2 days. The culture bottle was gently shaken every three to four days to expand the spread of the tachyzoites for easier observation. At around 15 days (with two or three additional drug selections carried out during this period), once a large number of tachyzoites had lysed, tachyzoite were injected intraperitoneally into mice, and then transferred back to HFF cells for two to three generations of cultivation. To ensure the genetic stability of the monoclonal strains, after blind transmission for 10 generations, genomic DNA was extracted and identified ME49 ∆*bfd2* strain by PCR. We designed specific primers separately, selected the1000 bp upstream (primers *bfd2*-PCR1-F/R) and downstream (primers *bfd2*-PCR2-F/R) of the ME49 *bfd2* coding DNA Sequence as homologous arms, and amplified homologous arms as PCR1 and PCR2. We selected the *bfd2* (1460 bp) sequence (primers *bfd2*-PCR3-F/R), as the CRISPR targeting site, and amplified it as PCR3. Transfectants were sorted for subcloned, and a lineage lacking BFD2 was verified by PCR. The correct monoclonal strain was identified as ∆*bfd2* for subsequent phenotype analysis.

### Plaque, invasion, and proliferation assays

The HFF (1 × 10^5^–5 × 10^5^/ml), ME49, and ME49Δ*bfd2* cells were sorted directly into 6-well and 12-well plates for subcloning. Invasion and proliferation assays were performed using HFF cells seeded in 12-well plates and grown to confluence. ME49 and ME49∆*bfd2* tachyzoites (1 × 10^5^ tachyzoites/well) were inoculated into the HFF cells. After removing non-invaded tachyzoites, 1 ml of DMEM medium supplemented with 1% FBS was added to each well, and the cells were incubated at 37°C with 5% CO_2_ for 24 hours. Post-incubation, the cells were stained, and the number of tachyzoites within 100 vacuoles was quantified using an inverted fluorescence microscope. The proportions of vacuoles containing >16, = 16, and ≤ 8 tachyzoites were calculated and recorded. Purified ME49 and ME49 ∆*bfd2* tachyzoites (100, 1000, and 10,000 tachyzoites/well) were inoculated into 6-well plates containing confluent HFF monolayers. The plates were incubated at 37°C with 5% CO_2_ for one week. After incubation, the cells were washed three times with PBS and fixed with 4% paraformaldehyde for 15 minutes, followed by another three washes with PBS. Each well was then stained with 2 ml of 2% crystal violet solution at room temperature for 15 minutes. Excess stain was removed by washing with PBS until plaques were clearly visible. Once the staining had dried, plaque analysis was performed.

### Western blotting

Cells were lysed using Radioimmunoprecipitation assay (RIPA) lysis buffer (300 μl, Beyotime Biotechnology) supplemented with protease inhibitor and phosphatase inhibitor cocktails (P8340, p5726, Sigma-Aldrich), and 1 mM of phenylmethylsulfonyl fluoride (PMSF). The lysates were centrifuged at 4°C (10,000 × *g* for 10 min) and the cellular debris was discarded. Proteins were loaded on 4–20% Mini-PROTEAN TGX Precast Gels (Bio-Rad, Hercules, CA, USA). The separated proteins were then transferred onto nitrocellulose membranes. The membranes were blocked in 5% skim milk and probed with the indicated primary antibodies at a dilution of 1:1,000, with Actin levels being used as a normalization control. The primary antibodies were used at the following dilutions: anti-NF-κB p65 (1:1,000), anti-pNF-κB p65 (1:1,000), anti-Stat3 (1:1,000), and anti- pStat3 (1:1,000).

### Quantitative real-time reverse transcription PCR (qRT-PCR)

The expression levels of *Il1b* (encoding Interleukin-1β (IL-1β)), *Ifng* (encoding interferon gamma) and *Cd86* (encoding CD86 molecule) were assessed in the ME49 and ME49Δ*bfd2* strains of *T. gondii* using qRT-PCR. Total RNA was prepared from isolated parasites using the TRNzol (Invitrogen, Carlsbad, CA, USA) reagent. Then cDNA was reverse transcribed from total RNA, with reverse transcriptase (RT) being excluded from the control reactions. For each sample, 2 μg of total RNA was reverse transcribed using a complementary DNA (cDNA) reverse transcription kit (Takara, Dalian, China). The cDNA was then subjected to qPCR. The comparative threshold cycle (2^−ΔΔCt^) method was used to evaluate the relative mRNA expression [17], and *Actb* (encoding β-actin (beta cytoskeletal actin)) levels were used as a normalization control.

### Enzyme-linked immunosorbent assay (ELISA)

On day 7 and day 30 post-infection, peripheral blood was collected from mice infected with ME49 and ME49Δ*bfd2 T. gondii*. The collected blood samples were left to stand at 4°C for 12 h and then centrifuged at 2,000 × *g* for 15 min to obtain the serum (supernatant), which was stored at −80°C. The levels of IL-1β, IFN-γ, and TNF-α in serum were detected using the corresponding ELISA kits, according to the manufacturer’s instructions (Bioswamp, Wuhan, China).

### Histological analysis

Brains of mice infected with *T. gondii* were fixed in 4% paraformaldehyde for subsequent experiments. Hematoxylin and eosin (H&E) staining was used to detect pathological damage. Nissl staining was performed to analyze neuronal structure. The staining data were analyzed using Image J software (NIH, Bethesda, MD, USA).

### RNA sequencing (RNA)-seq analysis

ME49 and ME49Δ*bfd2* parasites were allowed to invade BV2 cells in 10 cm dishes for 4 h, and then the cell monolayers were rinsed with PBS and switched to alkaline-stress media containing either 500 μM indole acetic acid (IAA) or PBS. After 72 h, the parasites were harvested and centrifuged (1,200 × *g* for 10 min). Total RNA was isolated using TRIzol (Thermo Fisher) and the RNA quality was assessed using a BioAnalyzer (AATI, Ames, IA, USA). Libraries were generated using RNA-seq and subjected to 150 × 150 paired-end sequencing on the NovaSeq 6000 platform (Illumina, San Diego, CA, USA). Gene Ontology (GO) annotation proteome results were derived from the database UniProt-GOA (http://www.ebi.ac.uk/GOA/). The protein sequences were searched to determine their potential function by using the domain database InterPro (http://www.ebi.ac.uk/interpro/). Biological process pathways or annotation were assessed using online service tools to describe proteins in the Kyoto Encyclopedia of Genes and Genomes (KEGG) database (http://www.genome.jp/kaas-bin/kaas_main, http://www.kegg.jp/kegg/mapper.html). An adjusted p-value (PADJ) of 0.05 was used as a statistical cutoff for differential expression. Data were deposited in the Genome Sequence Archive (Genomics, Proteomics & Bioinformatics 2021) in National Genomics Data Center (Nucleic Acids Res 2022), China National Center for Bioinformation/Beijing Institute of Genomics, Chinese Academy of Sciences repository with the identifier number: GSA: CRA022513.

### Immunofluorescence

Tissue slices were incubated with primary antibodies recognizing 4′,6-diamidino-2-phenylindole (DAPI) (1:200, Servicebio, China), ionized calcium-binding adapter molecule (IBA) (1:200, Affinity, Cincinnati, OH, USA), and neuronal nuclei antigen (NEUN) (1:200, Affinity) overnight at 4°C and placed in a sealed box with water on the bottom. The next day, slices were incubated in PBS and then incubated for 1 hour at room temperature with rhodamine-labeled secondary antibodies (1:200, ZSGB-BIO, Beijing, China). Following data collection, the densities of immunoreactive protein bands were computed using Image-Pro Plus 6.0 software (Media Cybernetics, Rockville, MD, USA).

### Apoptosis detection using a terminal deoxynucleotidyl transferase nick-end-labeling (TUNEL) assay

Brain tissues were collected from mice after infection with ME49 and ME49Δbfd2 *T. gondii*. Apoptosis in the brain tissues was detected using a TUNEL kit (Thermo Fisher) following the supplier’s guidelines. Detected apoptosis was recorded using Image J software.

### Elevated plus maze test (EPM) and Open field test (OFT)

The EPM and OFT were carried out according to published guidelines [18,19]. Changes in behavior were determined at the acute and chronic phases of infection with ME49 and ME49Δ*bfd2 T. gondii*. Changes in the number times the mice stood up and crossed the line (line crossing, moving distance, and speed were calculated to indicate the motor activity and exploratory behavior of mice). The EPM test and the OFT were used to measure the pre-anxiety-like behaviors of the mice. The following parameters were analyzed using EthoVision XT 11.5 software (Noldus Information Technology, Leesburg, VA, USA): The percentage of distance in the open arms, time in the open arms, speed in the open arms, total distance traveled, distance in the center, time in the center, and speed in the center.

### Statistical analysis

All quantitative data are reported as the mean ± SD. *P* < 0.05 was considered statistically significant. All statistical analyses and graph preparations were conducted using GraphPad Prism 9 (GraphPad Software Inc., San Diego, CA, USA).

## Results

### BFD2 aggravates the inflammatory response to *T. gondii* infection

**Fig 1:**
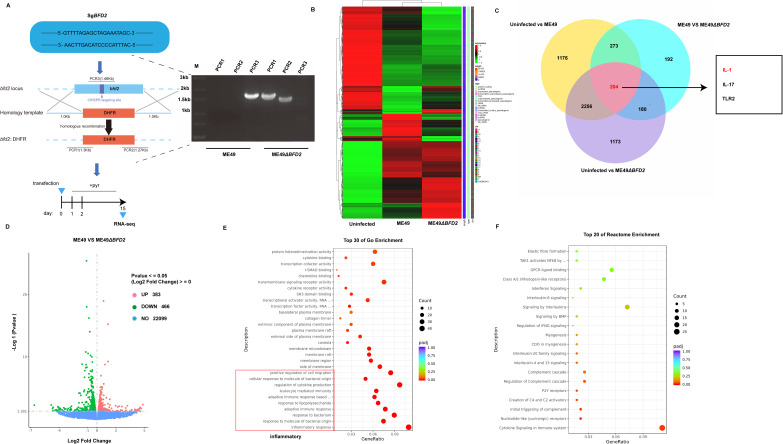
BFD2 aggravates the inflammatory response to *Toxoplasma gondii* infection. (A) Schematic diagram of ME49∆bfd2 knockout strain construction and screening of ∆bfd2 by PCR (M: Marker; PCR1, PCR2, and PCR3 amplification results of ME49 strain; PCR1, PCR2, and PCR3 amplification results of ME49 ∆bfd2 strain). (B) Heatmaps of the expression of DEGs with a fold change > 1.5 and false-discovery rate < 0.05 are shown for each sample. (C) A Venn diagram of commonly upregulated DEGs (n = 204) in the uninfected group, ME49 group, and ∆*bfd2* group. (D) Volcano map analysis showing 849 DEGs. (E, F) GO and KEGG analysis of DEGs identified in each group (n = 3) under *T. gondii* infection. Graphical representation of the enrichment of GO terms for commonly upregulated DEGs. The top 20 biological processes are shown.

Stable integration of the sgRNA is necessary for efficient clustered regularly interspaced short palindromic repeats (CRISPR)-mediated gene editing [20]. We designed the sgRNA targeting *bfd2* and transfected parasites with a CRISPR-associated protein 9 (CAS9)-expression plasmid carrying an acetyltransferase selectable marker, which was confirmed by sequencing (S1A Fig). Co-transfection of pSAG1::CAS9-U6::sgUPRT/sgBFD2 yielded ME49 and ME49∆*bfd2*-expressing parasites (the primers used are shown in S1 Table). Assays in cells infected with ME49 and ME49∆*bfd2* demonstrated that the levels of proliferation and plaque formation were significantly downregulated after deletion of *bfd2* (S1B, C Fig and S2 Table), suggesting that BFD2 could promote the survival of *T. gondii*. Next, we successfully constructed the ME49∆*bfd2* knockout strain, and established an *in vitro* ME49∆*bfd2* knockout strain infection model for RNA-seq using BV2 cells (Fig 1A). RNA-seq data was examined from samples of BV2 cells in the uninfected, ME49-infected, and ME49∆*bfd2*-infected groups, and based on the Pearson Correlation Coefficient, the correlation coefficient was close to 1 (S2A, B Fig). By comparing the RNA-seq data of uninfected, ME49, and ME49∆*bfd2* groups, the results suggested that in the model, in terms of biological processes, the differentially expressed genes (DEGs) were closely related to protein coding processes, transcribed processes, and biological regulation (Fig 1B). We identified 1,176 DEGs between the uninfected group and the ME49 group, 192 DEGs between the ME49 group and the ME49∆*bfd2* group, and 1,173 DEGs between the uninfected group and the ME49∆*bfd2* group. Additionally, 204 mRNAs (such as *Il1*, *Il17* and *TLR2*, et al.) were consistently expressed across all groups (Fig 1C). Volcano map analysis showed significant differences between the ME49 and ME49-∆*bfd2* groups, identifying 849 DEGs, including 383 upregulated genes and 466 downregulated genes (Fig 1D). Functional enrichment analysis of the DEGs showed a correlation with the inflammatory response (Figs 1E, F and S2C), indicating that BFD2 exacerbates the inflammatory response to *T. gondii* infection.

### BFD2 exacerbates the inflammation induced by acute stage *T. gondii* infection

**Fig 2:**
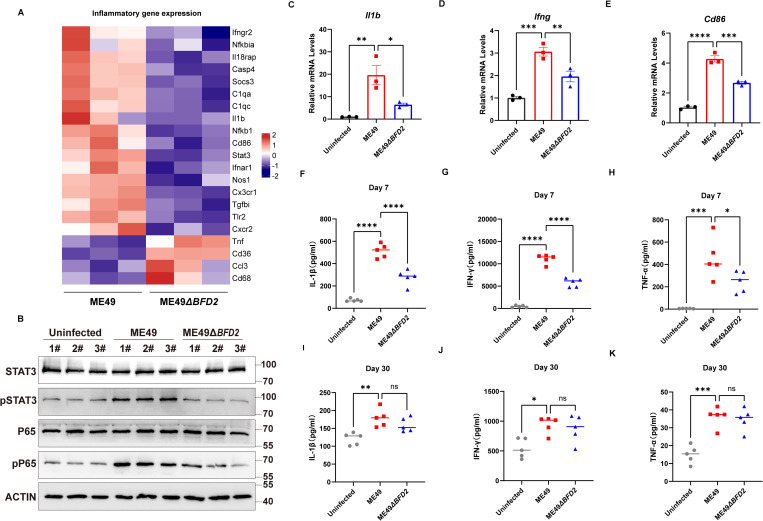
BFD2 exacerbates the inflammation induced in the acute stage of *T. gondii* infection. (A) Heat maps representing the RNA-sequencing data for inflammation-related DEGs in ME49 and ME49∆*bfd2* strain in BV2 cells (n = 3). (B) Western blotting detection of the indicated inflammation-related proteins in uninfected, ME49, and ME49∆*bfd2* strains (C-E) qRT-PCR determination of *Il1β*, *Ifng*, and *Cd86* expression. (F-K) Measurement of the IL-1β, IFN-γ and TNF-α protein levels in mice infected with ME49 and ME49∆*bfd2* strains in the acute and chronic stages of *T. gondii* infection (n = 5). Data are expressed as the mean ± SD of the indicated number of mice from one of three independent experiments.

Brief, RNA-seq analysis revealed that depletion of BFD2 abrogated the inflammatory factor signature, confirming that the induction of inflammatory factors observed during *T. gondii* infection is attributable to BFD2 activity (Fig 2A). We then extracted proteins and detected the levels of inflammation related indicators, including Stat3, pStat3, NF-κB p65 and pNF-κB p65, using western blotting. The results demonstrated that the levels of pStat3and pNF-κB p65 were significantly upregulated after ME49 infection, and downregulated after depletion of BFD2 (Fig 2B) Simultaneously, qRT-PCR was used to quantify *Il1b*, *Ifng*, and *Cd86* transcripts in cells infected with *T. gondii*. The results demonstrated low *Il1b*, *Ifng*, and *Cd86* mRNA expression during ME49 ∆*bfd2* strain infection (Fig 2C–E and S3–S5 Tables). To better understand the role of BFD2 in inducing inflammation during the different stages of *T. gondii* infection, we constructed acute stage and chronic stage models of ME49 and ME49∆*bfd2* infection in mice, together with uninfected mice, for *in vivo* analyses. Consistently, markedly decreased levels of IL-1β, IFN-γ and TNF-α were observed in the mice in the ME49∆*bfd2* group in the acute stage of *T. gondii* infection (Fig 2F–H and S6–S8 Tables). Interestingly, the levels of these inflammatory indicators did not change significantly during the chronic stage of *T. gondii* infection (Fig 2I–K and S9–S11 Tables), illustrating that BFD2 exacerbates the inflammation induced during the acute stage, but not the chronic stage, of *T. gondii* infection.

### BFD2 mediates brain inflammation and apoptosis induced by the acute stage of *T. gondii* infection

**Fig 3:**
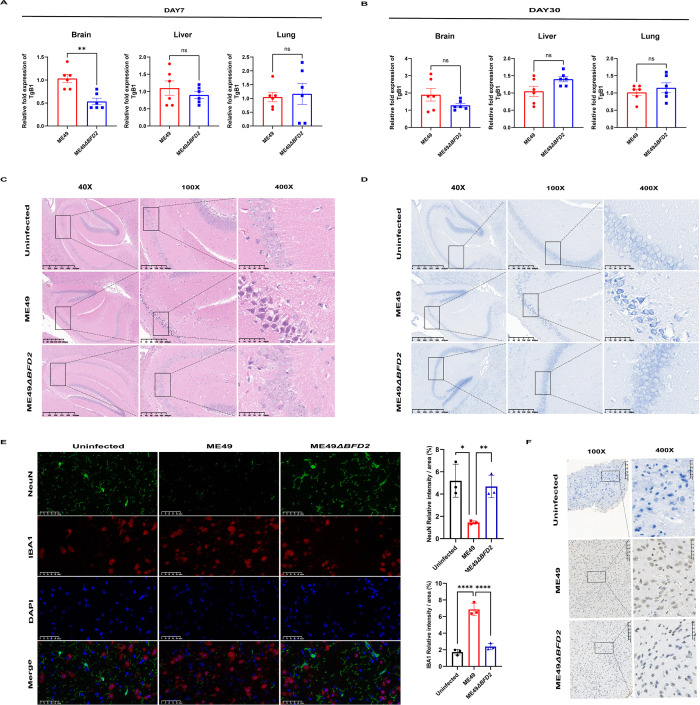
BFD2 mediates brain inflammation induced in the acute stage of *T. gondii* infection. (A, B) Measurement of *T. gondii* loads in the brain, liver, and lungs of ME49- and ME49∆*bfd2*-infected mice (n = 6). (C, D) Representative imaging showing HE and Nissl staining of brain damage in uninfected, ME49-infected, and ME49∆*bfd2-*infected mice during the acute stage of *T. gondii* infection. (E, F) Immunofluorescence and TUNEL staining in mice during the acute stage of *T. gondii* infection (magnification 40 × , scale bar = 625 μm; magnification 100 × , scale bar = 200 μm; magnification 400 × , scale bar = 50 μm, n = 3).

To further explore of the sites affected by *T. gondii* infection-induced inflammation, we constructed model acute stage and chronic stage ME49 and ME49∆*bfd2* infection in mice and compared them with uninfected mice. The *T. gondii* Bradyzoite-specific protein 1 (TgB1) levels were significantly decreased in the brain, but showed no significant changes in liver and lung, when ME49-infected mice were compared with ME49∆*bfd2*-infected mice during the acute stage (Fig 3A and S12 Table). In the chronic stage, TgB1 levels in the brain, liver, and lung showed no differences between the ME49-infected mice and ME49∆*bfd2*-infected mice (Fig 3B and S13 Table). In the acute stage of *T. gondii* infection, we firstly examined changes to neuronal morphology and Nissl bodies using HE and Nissl staining. We observed that the brain tissue of the ME49-infected group had irregular and fragmented neuronal cell membranes with fewer nuclei compared with the control group and the ME49∆*bfd2*-infected group (Fig 3C, D). In the chronic stage of *T. gondii* infection, there was no significant difference in neuronal morphology and Nissl bodies when *bfd2* was knocked out (S3 and S4 Figs). We also observed the expression levels of neuronal nuclei antigen (NEUN) and ionized calcium-binding adapter molecule 1 (IBA1), which are makers of microglia and neuronal cells, respectively. The results showed that the number of microglia cells increased and neuronal cells decreased significantly in the ME49 group compared with that in the ME49∆*bfd2* group (Fig 3E and S14 Table). Focused KEGG analysis showed that apoptotic signals were increased during *T. gondii* infection (S5 Fig). Furthermore, compared with that in the control group and ME49∆*bfd2* group, the results of the TUNEL assay revealed that the level of apoptosis was higher in the ME49 group (Fig 3F). These results showed that BFD2 activates and mediates brain inflammation and apoptosis induced during the acute stage of *T. gondii* infection.

### BFD2 impacts pre-anxiety-like behaviors in the acute stage of *T. gondii* infection

**Fig 4:**
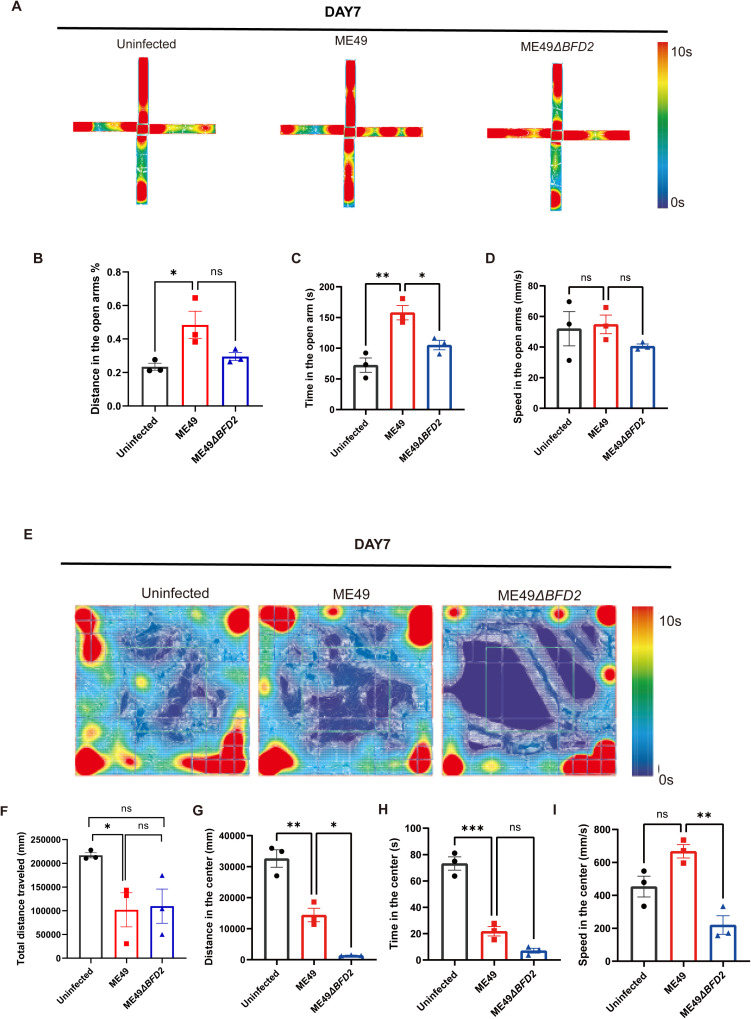
BFD2 impacts pre-anxiety-like behaviors in the acute stage of *T. gondii* infection. (A-D) Elevated plus maze test in mice infected with ME49 and ME49∆*bfd2* in the acute stage of *T. gondii* infection. N = 3 per group. (E-I) Open field test in mice infected with ME49 and ME49∆*bfd2* in the acute stage of *T. gondii* infection. N = 3 per group. Data are expressed as means ± SEM.

After establishing the animal model, we detected the spontaneous locomotor activity and pre-anxiety-like behavior using the EPM and OFT on day 7. By EPM, compared with the uninfected group and ME49∆*bfd2* group, the ME49 group mice increased the percentage of distance in the open arms (%) and increased the amount of time that they spent on the open arms (s) after depletion of BFD2 (Fig 4A–C and S15 and S16 Tables). Meanwhile, for the uninfected group, ME49 group and ME49∆*bfd2* group, the speed in the open arms (mm/s) showed no significant differences (Fig 4D and S17 Table). We also detected the anxiety-like behavior by OFT. The results showed the total distance traveled (mm), distance in the center (mm), and time in the center (s) were significantly decreased in the ME49 group, and the total distance traveled (mm) showed no significant changes in the ME49∆*bfd2* group (Fig 4E–H and S18–S20 Tables). Consistent with the EPM results, in the uninfected group, ME49 group, and ME49∆*bfd2* group, the speed in the center (mm/s) was not significant different (Fig 4I and S21 Table). All these results suggested that BFD2 impacts pre-anxiety-like behaviors in mice during the acute stage of *T. gondii* infection.

### BFD2 shows no impact on anxiety in chronic *T. gondii* infection

**Fig 5:**
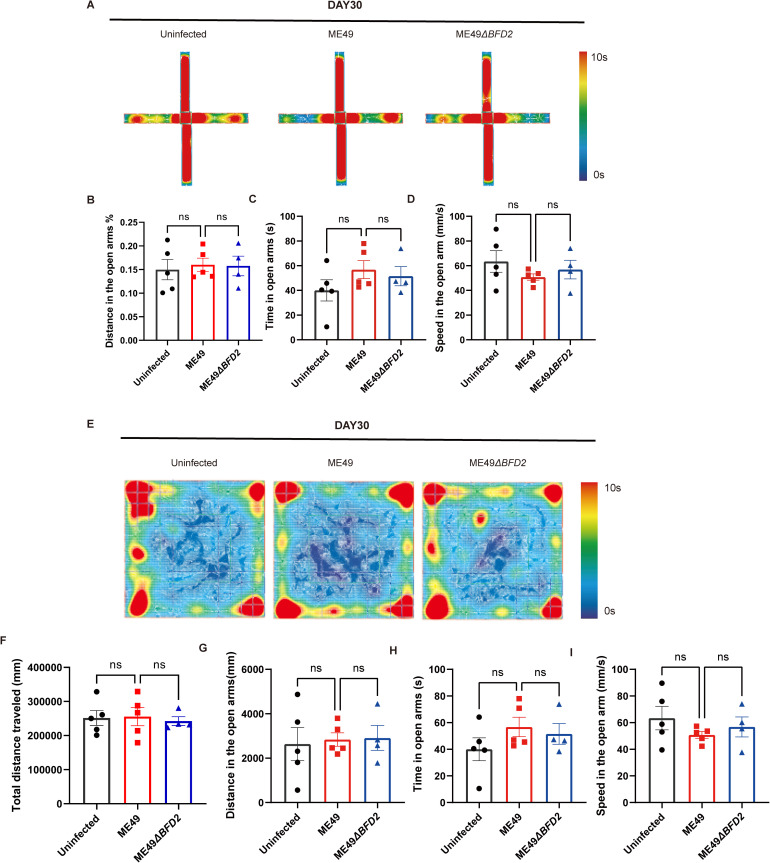
BFD2 regulates anxiety-like behaviors induced by acute-stage *T. gondii* infection. (A-D) elevated plus maze test in mice infected with ME49 and ME49∆*bfd2* in the chronic stage of *T. gondii* infection. N = 5/group. (E-I) Open field test in mice infected with ME49 and ME49∆*bfd2* in the chronic stage of *T. gondii* infection. N = 5/group. Data are expressed as means ± SEM.

We further detected anxiety-like behavior the chronic stage of *T. gondii* infection by EPM and OFT. Among the uninfected group, ME49 group, and ME49∆*bfd2* group, the percentage of distance in the open arms (%), time in the open arms (s), speed in the open arms (mm/s), total distance traveled (mm), distance in the center (mm), time in the center (s), and speed in the center (mm/s) were not significantly different (Fig 5A–I and S22–S28 Tables). The results indicated no differences in behavioral performance in the mice during the chronic stage of *T. gondii* infection.

## Discussion

*T. gondii* is a significant food-borne zoonotic parasite, and approximately one-third of the global human population are positive for *T. gondii* antibodies [21]. Although infected animals and people can survive for a long time carrying these cysts, the active replication of the proliferative form (tachyzoites) can lead to serious and potentially fatal diseases. During the acute stage of infection, *T. gondii* tachyzoites spread throughout the body and cause pathological symptoms *via* host cell lysis, and some tachyzoites produce bradyzoites, which are encapsulated in brain and muscle tissue [22]. Most infections are controlled by the host immune response. However, during periods of weakened immune function, latent cysts might reactivate [23]. Encystation, in which the tachyzoite-to-bradyzoite transition occurs, is a key feature of *T. gondii* persistence. Research reveals that both BFD1 and AP2 transcription factors are important for the tachyzoite-to-bradyzoite transition [11,24]. AP2, one of the candidate molecules that interact with BFD1, jointly regulates the tachyzoite-to-bradyzoite transition—a process in which BFD1 is essential [11,12]. Furthermore, BFD2, a cytoplasmic RNA-binding protein of the CCCH zinc-finger family, transcriptionally activates BFD1 and directly induces its expression [25]. In addition to chronic infections caused by slow colonization of hosts, acute infections caused by tachyzoites are also worthy of research attention. A recent study suggested that BFD2 is a key switch regulating the differentiation of *T. gondii*, representing a transcription factor required to control the transition from tachyzoites to bradyzoites [12]. Although that study demonstrated that the absence of BFD2 prevents the formation of cysts, which in turn leads to the inability of *T. gondii* to survive in the long term, it also observed the presence of *T. gondii* in mouse brain tissue after 45 days of infection in the absence of BFD2 [12]. The role of BFD2 in inducing inflammation remains unclear. Notably, a study found that the protein phosphatase 2A, which regulates the phenotypic switching between tachyzoites and bradyzoites in *T. gondii*, can induce acute infection [26]. Given the above findings, we suggested that BFD2 might cause host damage during the acute stages of *T. gondii* infection.

Although BFD2 promotes the transformation of bradyzoites, it is unclear whether it is involved in inflammation, apoptosis, and pre-anxiety during the acute stage of *T. gondii* infection. Herein, we successfully constructed the ME49∆*bfd2* strain of *T. gondii* and established *in vivo* and *in vitro* infection models. We revealed that BFD2 plays a crucial role in the acute stage of *T. gondii*-associated inflammation, apoptosis, and pre-anxiety in mouse brains. We also showed that acute *T. gondii* infection induced inflammation. This is consistent with the results of previous studies [27,28]. Moreover, we reported that *T. gondii*-associated inflammation is induced by the BFD2-mediated phenotype switching between tachyzoites and bradyzoites.

Studies have shown that inflammation is associated with neuron cells apoptosis [29–32]. For example, induced neuroinflammation and neurotoxicity are induced through the NF-kB/STAT3/ERK and mitochondria-mediated apoptosis pathway [33], inflammation and neuronal oxidative injury are induced by NF-κB phosphorylation [34], and neuroinflammation and neuronal apoptosis are induced *via* the PI3K/Akt pathway [35]. *T. gondii* infection induces an inflammatory response, neuronal cell death, and the infiltration and activation of microglia, thereby regulating central nervous system infection-related immunity [36]. Herein, we report that BFD2 increases the levels of pStat3 and pNF-κB p65 during *T. gondii* infection. In addition, *T. gondii* infection triggers host cell mitochondrial dysfunction, manifested as reduced mitochondrial membrane potential, ROS accumulation, and cytochrome C release, which activate the caspase cascade to execute apoptotic programs [37–39]. We showed that BFD2 mediated *T. gondii* infection-induced neuron damage in the hippocampus, which was characterized by neuron cell apoptosis. However, the exact regulatory mechanisms warrant additional exploration. Studies have demonstrated that the symptoms of neurological disorders in patients’ brains are caused by neuroinflammation, which leads to increased neuronal cell death [40–42]. Thus, we propose that BFD2 exacerbates brain inflammation and apoptosis, which might impact mouse behavior during the acute stage of *T. gondii* infection.

Furthermore, various studies have shown that *T. gondii* is associated with anxiety-like behaviors in mice [14–16,43–45]. Moreover, anxiety-like behaviors are mainly caused by chronic *T. gondii* infection [46–48]. Interestingly, we showed that BFD2 could induces pre-anxiety-like behaviors in mice with acute *T. gondii* infection. The central nervous system is strongly associated with anxiety, and neuroinflammation is regarded as the most important neuropathological basis for anxiety [49,50]. In addition, the pro-inflammatory cytokines TNF-α and IL-1β have been reported to induce anxiety-like behaviors [51,52]. Correspondingly, we report that BFD2 significantly elevated the mRNA expression levels of *Il1b*, *Ifng*, *Tnfa*, and *Cd86* in mice with acute *T. gondii* infection. These results suggest that BFD2 regulates pre-anxiety-like behaviors in mice. However, our study was limited by the act that it did not provide a detailed explanation of the specific mechanism of BFD2 regulation. BFD2-mediated brain inflammation, apoptosis, and pre-anxiety-like behaviors deserve more detailed investigations in the future, which might provide hitherto unknown mechanistic insights.

## Supporting information

S1 Table List of PCR primer sequences used for bfd2 genome editing *via* CRISPR/Cas9. (XLSX)

S2 Table The plaque assay results of the ME49 and ME49∆*bfd2* strain *in vitro.* (XLSX)

S3 Table qRT-PCR detection of *Il1b* transcripts in the different stages of *T. gondii* infection. (XLSX)

S4 Table qRT-PCR detection of *Ifng* transcripts in the different stages of *T. gondii* infection. (XLSX)

S5 Table qRT-PCR detection of *Cd86* transcripts in the different stages of *T. gondii* infection. (XLSX)

S6 Table The level of Il-1β detected using ELISA in host tissue during acute *T. gondii* infection. (XLSX)

S7 Table The level of IFN-γ detected using ELISA in host tissue during acute *T. gondii* infection. (XLSX)

S8 Table The level of TNF-α detected using ELISA in host tissue during acute *T. gondii* infection. (XLSX)

S9 Table The level of Il-1β detected using ELISA in host tissue during chronic *T. gondii* infection. (XLSX)

S10 Table The level of IFN-γ detected using ELISA in host tissue during chronic *T. gondii* infection. (XLSX)

S11 Table The level of TNF-α detected using ELISA in host tissue during chronic *T. gondii* infection. (XLSX)

S12 Table The expression level of *TgB1* using qRT-PCR in host tissue during acute *T. gondii* infection. (XLSX)

S13 Table The expression level of *TgB1* using qRT-PCR in host tissue during chronic *T. gondii* infection. (XLSX)

S14 Table The expression level of NEUN and IBA1 using immunofluorescence (Column chart) by infected *T. gondii.* (XLSX)

S15 Table Elevated plus maze test (percentage of distance in the open arms) in mice infected with ME49 and ME49∆*bfd2 T. gondii* in the acute stage. (XLSX)

S16 Table Elevated plus maze test (time in open arms) in mice infected with ME49 and ME49∆*bfd2 T. gondii* in the acute stage. (XLSX)

S17 Table Elevated plus maze test (speed in open arms) in mice infected with ME49 and ME49∆*bfd2 T. gondii* in the acute stage. (XLSX)

S18 Table Open field test (total distance traveled) in mice infected with ME49 and ME49∆*bfd2 T. gondii* in the acute stage. (XLSX)

S19 Table Open field test (distance in center) in mice infected with ME49 and ME49∆*bfd2 T. gondii* in the acute stage. (XLSX)

S20 Table Open field test (time in center) in mice infected with ME49 and ME49∆*bfd2 T. gondii* in the acute stage. (XLSX)

S21 Table Open field test (speed in center) in mice infected with ME49 and ME49∆*bfd2 T. gondii* in the acute stage. (XLSX)

S22 Table Elevated plus maze test (percentage of distance in the open arms) in mice infected with ME49 and ME49∆*bfd2 T. gondii* in the chronic stage. (XLSX)

S23 Table Elevated plus maze test (time in open arms) in mice infected with ME49 and ME49∆*bfd2 T. gondii* in the chronic stage. (XLSX)

S24 Table Elevated plus maze test (speed in open arms) in mice infected with ME49 and ME49∆*bfd2 T. gondii* in the chronic stage. (XLSX)

S25 Table Open field test (total distance traveled) in mice infected with ME49 and ME49∆*bfd2 T. gondii* in the chronic stage. (XLSX)

S26 Table Open field test (distance in center) in mice infected with ME49 and ME49∆*bfd2 T. gondii* in the chronic stage. (XLSX)

S27 Table Open field test (time in center) in mice infected with ME49 and ME49∆*bfd2 T. gondii* in the chronic stage. (XLSX)

S28 Table Open field test (speed in center) in mice infected with ME49 and ME49∆*bfd2 T. gondii* in the chronic stage. (XLSX)

S1 Fig Construction and identification of the ME49∆*bfd2* knockout strain. (DOCX)

S2 Fig Sample correlation and KEGG analysis. (DOCX)

S3 Fig HE staining of a brain in the chronic stage of *T. gondii* infection. (DOCX)

S4 Fig Nissl staining of a brain in the chronic stage of *T. gondii* infection. (DOCX)

S5 Fig KEGG analysis of identified DEGs. (DOCX)
