# Supplementary material for: BFD2 mediates inflammation, apoptosis, and pre-anxiety-like behaviors induced by acute Toxoplasma gondii infection
Source: PLoS Negl Trop Dis. 2025 Sep 4;19(9):e0013428. doi: 10.1371/journal.pntd.0013428 (PMC12410722; doi:10.1371/journal.pntd.0013428)
Supplement: S1 Fig — (DOCX) [file pntd.0013428.s029.docx]

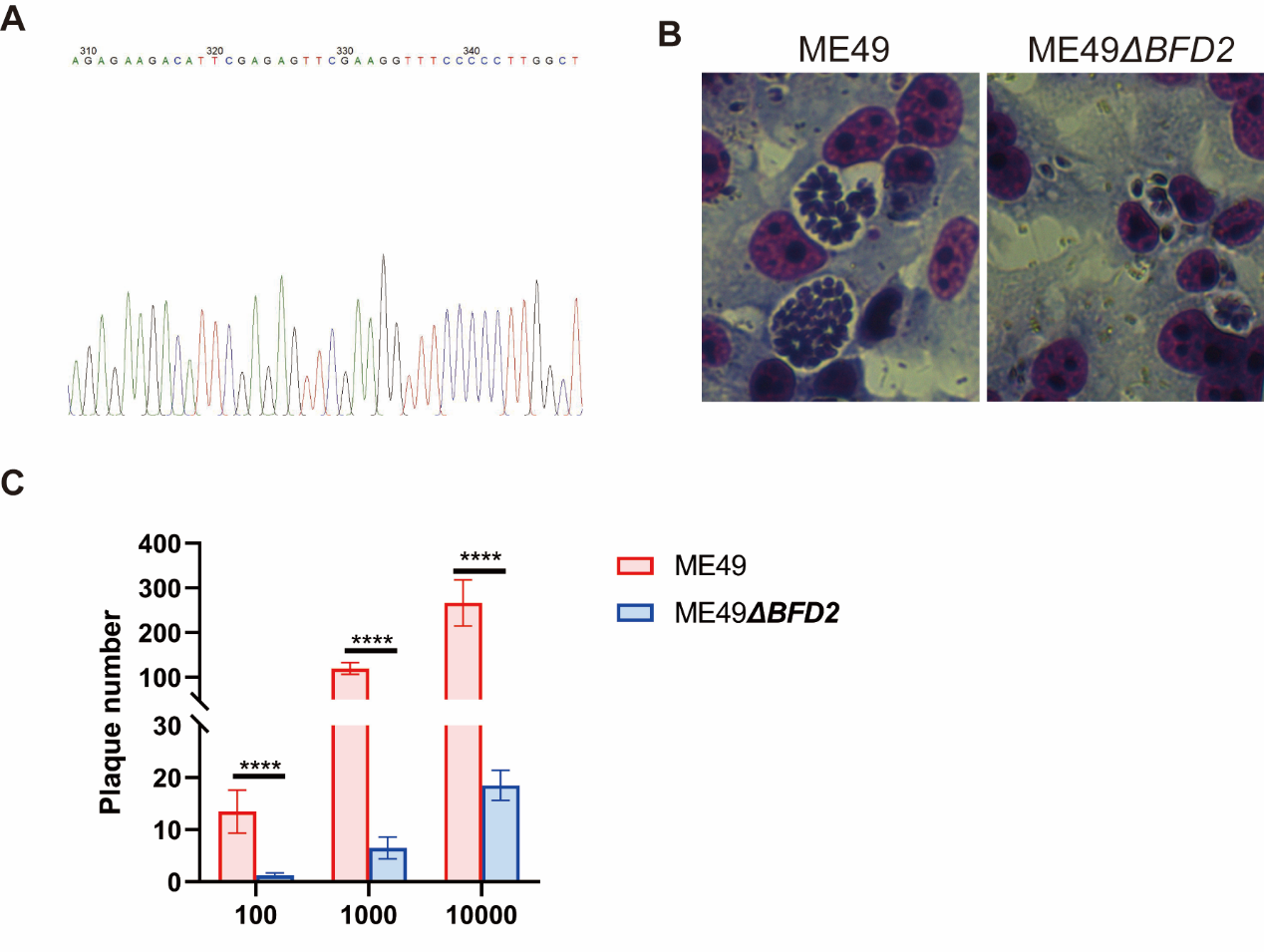


**S1 Fig. Construction and identification of the ME49∆*bfd2* knockout strain** (A) Chromatogram showing the presence of the ME49∆*bfd2* strain. (B, C) Plaque and proliferation assay results of the ME49 and ME49∆*bfd2* strains *in vitro* (n = 4).
