# Supplementary material for: BFD2 mediates inflammation, apoptosis, and pre-anxiety-like behaviors induced by acute Toxoplasma gondii infection
Source: PLoS Negl Trop Dis. 2025 Sep 4;19(9):e0013428. doi: 10.1371/journal.pntd.0013428 (PMC12410722; doi:10.1371/journal.pntd.0013428)
Supplement: S2 Fig — (DOCX) [file pntd.0013428.s030.docx]

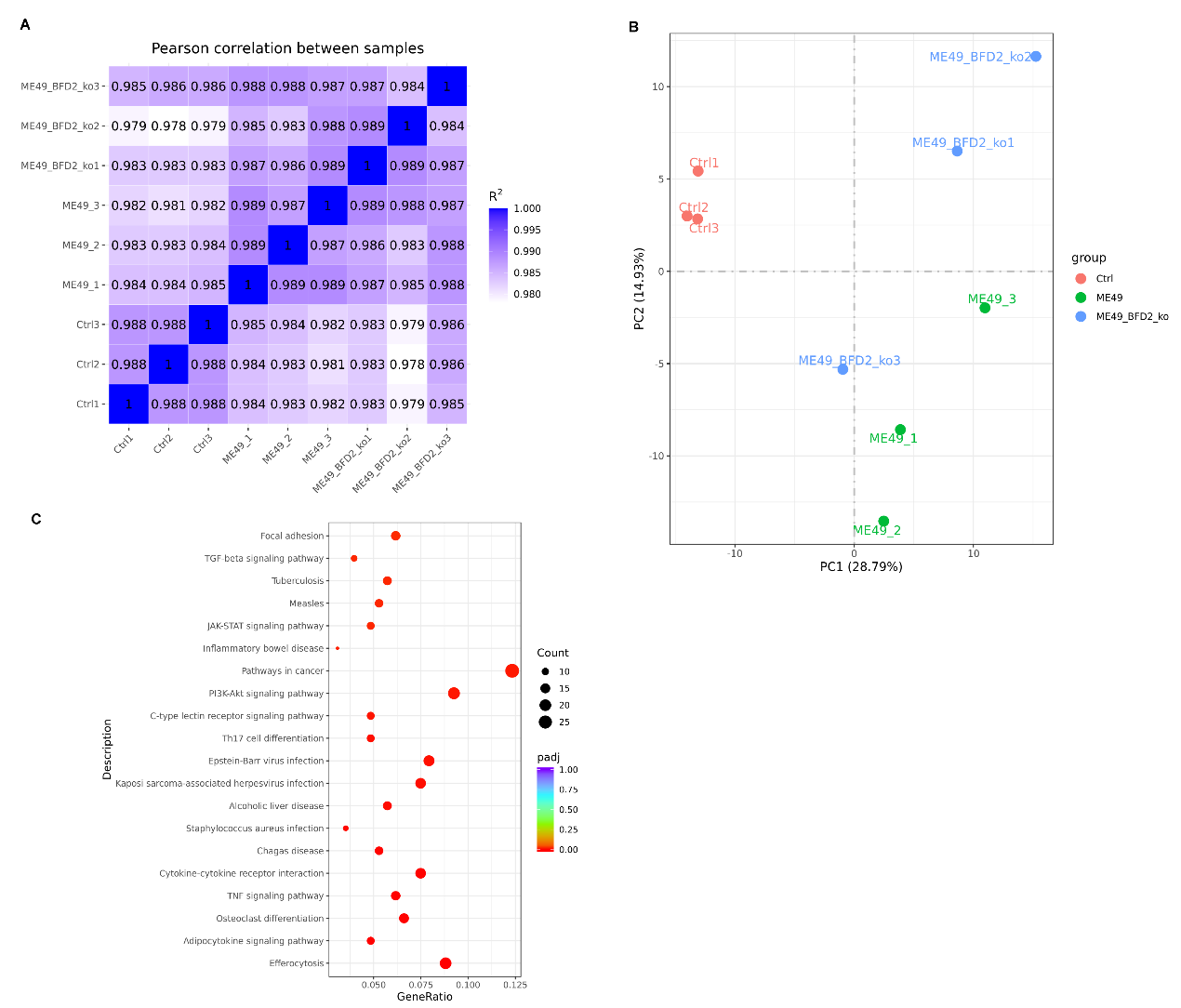


**S2 Fig. Sample correlation and KEGG analysis** (A, B) Correlation and principal component analysis of expressed genes in the uninfected groups, and mice infected the ME49 and ME49∆*bfd2*. R^2^ = 0.8~1 was used as the selection criterion. (C) KEGG enriched pathways for DEGs related to inflammation (n = 3).
