# Supplementary material for: BFD2 mediates inflammation, apoptosis, and pre-anxiety-like behaviors induced by acute Toxoplasma gondii infection
Source: PLoS Negl Trop Dis. 2025 Sep 4;19(9):e0013428. doi: 10.1371/journal.pntd.0013428 (PMC12410722; doi:10.1371/journal.pntd.0013428)
Supplement: S3 Fig — (DOCX) [file pntd.0013428.s031.docx]

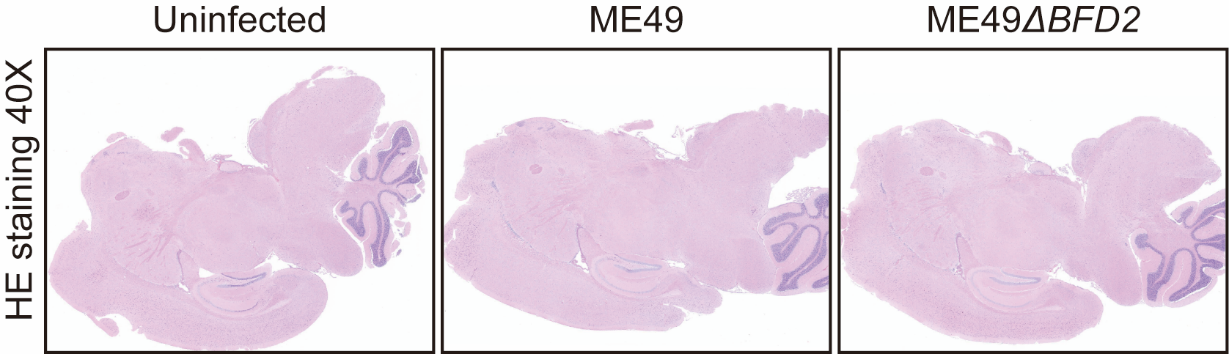


**S3 Fig. HE staining of a brain in the chronic stage of *T. gondii* infection**

Representative imaging showing HE staining of brain damage in uninfected, ME49, and ME49∆*bfd2*infected in mice during the chronic stages of *T. gondii* infection (magnification 40×, scale bar = 625 μm, n =3).
