# Supplementary material for: BFD2 mediates inflammation, apoptosis, and pre-anxiety-like behaviors induced by acute Toxoplasma gondii infection
Source: PLoS Negl Trop Dis. 2025 Sep 4;19(9):e0013428. doi: 10.1371/journal.pntd.0013428 (PMC12410722; doi:10.1371/journal.pntd.0013428)
Supplement: S5 Fig — (DOCX) [file pntd.0013428.s033.docx]

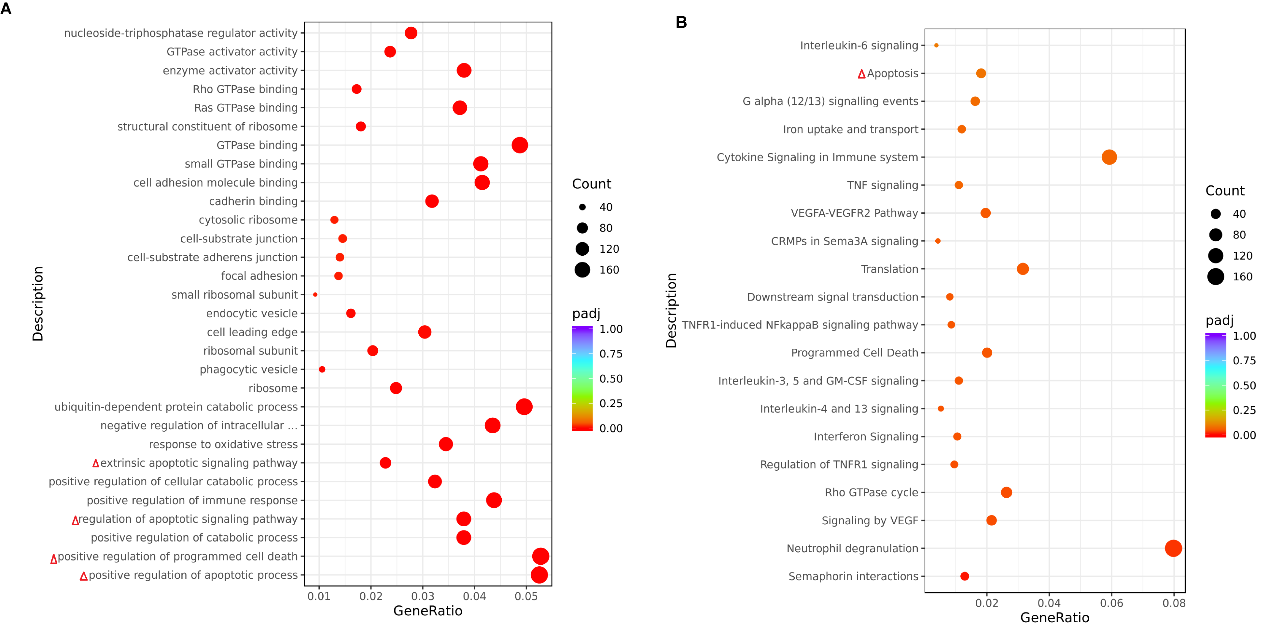


**S5 Fig. KEGG analysis of identified DEGs** (A, B) 'Rich' refers to the ratio of the number of DEGs enriched in the pathway to the number of DEGs annotated. PADJ generally ranges from 0 to 1, red indicates close to zero and significant enrichment, blue indicates close to 1 and insignificant enrichment. KEGG enriched pathways for DEGs related to apoptosis are shown (n = 3).
